# Correlation between kinetic and kinematic measures, clinical tests and subjective self-evaluation questionnaires of the affected upper limb in people after stroke

**DOI:** 10.3389/fnins.2023.1264513

**Published:** 2023-12-04

**Authors:** Ronnie Baer, Ronit Feingold-Polak, Daniel Ostrovsky, Ilan Kurz, Shelly Levy-Tzedek

**Affiliations:** ^1^Recanati School for Community Health Professions, Department of Physical Therapy, Faculty of Health Sciences, Ben-Gurion University of the Negev, Be'er Sheva, Israel; ^2^Herzog Medical Center, Jerusalem, Israel; ^3^Clinical Research Center, Soroka University Medical Center, Ben-Gurion University of the Negev, Be'er Sheva, Israel; ^4^Zelman Center for Neuroscience, Ben-Gurion University of the Negev, Beer-Sheva, Israel; ^5^Freiburg Institute for Advanced Studies (FRIAS), University of Freiburg, Freiburg, Germany

**Keywords:** stroke, kinematics, kinetics, clinical test, self-evaluation questionnaires

## Abstract

**Introduction:**

Assessment of stroke recovery should include multiple sources of information in order to obtain a complete understanding of the individual’s rehabilitation progress. Self-evaluation questionnaires’ scores do not always correspond to the scores of commonly used clinical evaluation tools. The purpose of this study was to assess the relationship between self-evaluation questionnaires, clinical tests, and kinematic and kinetic analyses of the affected upper limb after stroke, and to determine the correlation between these measures and self-reported general function 2–4 years after the stroke.

**Methods:**

Twenty-six subjects recovering from stroke were included in the study. Spearman’s correlation coefficient was used to measure the correlation between Stroke Impact Scale (SIS), Motor activity Log (MAL), Fugl-Meyer Assessment (FMA) and Action Reach Arm Test (ARAT) scores, and kinematic and kinetic analyses. A logistic regression was used to assess the extent to which these measures may predict the participants’ functional self-reported status 2–4 years post stroke.

**Results:**

Sections regarding hand function, hand force and general ADL of the self-evaluation questionnaires correlated with kinematic variables. However, only questionnaires that focus on hand function correlated with clinical tests. Mean and maximal hand velocity had the strongest correlations with self-evaluation questionnaires and with the clinical tests, more than other kinematic variables. Self-evaluation questionnaires and clinical tests were found to be correlated with hand kinetic metrics force-to-time ratio and number of force peaks. SIS hand force domain, mean velocity and maximal velocity predicted self-reported general function 2–4 years after the stroke.

**Conclusion:**

Self-evaluation questionnaires should be considered for wider use in the clinical evaluation of a patient’s stroke recovery, since they add important information on the individual’s functional status, which is not reflected in the clinical tests.

## 1 Introduction

Neglecting to account for the patient’s perspective – that is, how they perceive their rehabilitation progress – in all phases of post-stroke rehabilitation is inadvisable: A discrepancy between clinical evaluation scores and self-evaluation reports (Dromerick et al., 2006; van Delden et al., 2013; Persson et al., 2015; Banina et al., 2017; Essers et al., 2019; Cameron et al., 2020; Essers et al., 2021) suggests that the clinical evaluation process may have a blind spot. Post-stroke individuals often find their functional abilities unsatisfactory, even while their scores on clinical tests are high or even maximal (Han et al., 2013; Stewart and Cramer, 2013). In addition, when testing the use of the affected hand post stroke in everyday activities with accelerometers (Waddell et al., 2017), no change was found in the use of the involved hand following intervention, even though the clinicians rated significant change in hand function on clinical scores. It is thus important to find complementary tools to the clinical evaluation scales, which can provide a wider picture of the status of the patient. Kinetic and kinematic measures may be able to provide more nuanced data on movement quality, but specialized equipment for measuring functional movements may not be available in all clinics; our goal in the current work was thus to test whether self-evaluation questionnaires – which are simple to use and are low-cost to implement – can help, alongside clinical tests, to detect the functional difficulties which are not reflected in the clinical tests alone.

### 1.1 Stroke sequelae

After stroke, more than 80% of survivors are left with various deficits which largely affect their ability to perform daily activities; from sensorimotor impairments to psychological and neuropsychiatric disturbances (Broeks et al., 1999; Ferro et al., 2016; Waller et al., 2016; Winstein et al., 2016; Perrain et al., 2020).

### 1.2 Importance of recovery assessment in stroke rehabilitation

According to the International Classification of Function (ICF) model, when assessing an individual’s general recovery, body functions and structures, activity limitations and participation restrictions should all be evaluated in order to procure the full picture of the individual’s rehabilitation (Üstün et al., 2003). In today’s clinical setting, clinical tests are a common evaluation tool for post-stroke patients. Clinical tests are administered by the patient’s therapist and are considered valid and reliable for recovery evaluation (Platz et al., 2005; Lang et al., 2013; Lundquist and Maribo, 2017). While clinical tests are considered robust assessment tools, they are administered in clinical settings, which means that they are not capable of evaluating individuals in their daily environment. Furthermore, scores on clinical tests cannot take into account the patient’s perspective. Subjective self-evaluation scales, also known as Patient Reported Outcome Measures (PROMS), provide important information regarding the individual’s challenges in everyday life as they perceive them, which are not necessarily observable to external evaluators (Stewart and Cramer, 2013; Banina et al., 2017; Katzan et al., 2017). For example, PROMS can provide information about tasks that the individual struggles to perform at home and cannot be observed in the clinical setting such as taking a shower or driving a car. They are relatively simple to use and low-cost to implement. Alongside the clinical tests and the PROMS, objective measures, such as kinematic and kinetic analyses are sensitive to small changes and provide an accurate, unbiased evaluation, which is highly valuable in assessing progress. These tools can also quantify the quality of movement, using parameters such as hand velocity and jerk (Kashi et al., 2020; Feingold-Polak et al., 2021a,b); this way, they provide an extensive estimation of the individual’s motor ability (Subramanian et al., 2010; Levin et al., 2016), but may be expensive and require technical knowledge to implement in the clinic. Self-evaluation scores (PROMS), observed functional ability (clinical tests) and kinematic and kinetic analyses are all important for the assessment of the rehabilitation process (Gladstone et al., 2002; Chen et al., 2012; Stewart and Cramer, 2013; Schwarz et al., 2019). Surprisingly, there is no uniform correspondence between their outcomes.

### 1.3 Mismatch between clinical tests and self-evaluation questionnaires

Recent studies emphasize the importance of patient-centered care and patient motivation in the rehabilitation process (Danks et al., 2016; French et al., 2016; Wulf and Lewthwaite, 2016). Nowadays, since PROMS are not in wide use in the clinical field, the patient’s perception of their rehabilitation is usually not reflected in the commonly used evaluation tools. However, it highly affects the patient’s quality of life and can be used to improve both the care of a specific individual and of a general diagnostic group (Winstein and Varghese, 2018). A few previous works found a discrepancy between observed clinical evaluation tests and self-evaluation scales in a post-stroke population (Banina et al., 2017; Essers et al., 2019). Studies found that stroke survivors report limited hand use, even in cases in which the motor recovery of the affected hand is full, as indicated by different clinical tests and tools (Han et al., 2013; Stewart and Cramer, 2013). Self-evaluation questionnaires provide meaningful information that cannot be obtained through clinical external evaluation, and they were found to be more sensitive to changes in the patient’s status (Katzan et al., 2017). The underlying causes of this mismatch – between clinical scores and actual hand use – are not yet clear. It is possible that kinetic and kinematic measures might offer additional information to that available through clinical tests, thus uncovering the motor deficits which are not reflected in clinical tests, yet limit everyday function. There are thus at least three tools that complement each other in reflecting the functional status of the patient: the clinical tests, the kinematic and kinetic measurements and the self-evaluation questionnaires. Ideally, they would all be used in the clinic together. However, since highly specialized equipment for measuring kinetics and kinematics is usually not available in clinical practice, it would be helpful to understand the extent of overlap between the kinetic and kinematic functioning of the hand and the self-evaluation questionnaires, since the latter is more straightforward to use in the clinic; We set out to test whether self-evaluation questionnaires – which are easy and cheap to use – can help detect the functional difficulties which are not reflected in clinical tests.

These days, self-evaluations questionnaires are not commonly used in stroke rehabilitation clinical practice. One study which examined the correlation between a self-evaluation scale and a kinematic analysis of the upper limb (UL) at five time points after stroke found that the correlation between the two improved with time, but only in certain aspects of the kinematic analysis (Hussain et al., 2020); For example, the correlation between movement time and the score on the ABILHAND questionnaire improved with time, but peak velocity showed low correlation with ABILHAND at all time points. In addition, the kinematic task participants were asked to perform was not an everyday, functional activity; they were instructed to point a stylus at different targets in a virtual reality (VR) environment. The functionality of a kinematic measurement task is highly important since only functional, everyday tasks can represent the real-life activity of an individual (Bayona et al., 2005; Hubbard et al., 2009). Therefore, it is impossible to deduce from tasks that are not routinely performed in the patient’s home environment on their actual functional abilities (Winstein et al., 2016).

Further investigation is needed to thoroughly comprehend the relationship between clinical tests, different self-evaluation scales and a *functional* kinetic and kinematic analysis of the affected UL after stroke. Understanding which domains of the self-evaluation scales and which parameters of the kinetic and kinematic analysis correlate could help clinicians understand which possible impairments should be treated in cases of low recovery perception, which cannot be explained by standard clinical tests (Metrot et al., 2013; van Dokkum et al., 2014).

The self-evaluation questionnaires provide a patient-perspective-centered evaluation tool. We wish to understand whether the commonly used clinical tests are sufficient, or whether the self-evaluation questionnaires measure an aspect of the individual’s rehabilitation that cannot be reflected in other assessment tools.

The aims of this study were of threefold: first, to explore the importance of self-evaluation questionnaires as part of the clinical patient evaluation process; second, to determine which functional kinematic and kinetic variables correlate with patients’ clinical assessment and self-perceived recovery; Third, to compare the ability of the different assessment measures to predict self-perceived functional status 2–4 years post-stroke. This information can be utilized by researchers in future studies and by clinicians when choosing which assessment measures to use in their evaluation process. We hypothesize that there will be a stronger relationship between kinematic and kinetic variables and *self-evaluation questionnaires* than between kinematic and kinetic variables and *clinical tests*. In addition, we hypothesize self-evaluation questionnaires and kinematic and kinetic variables will predict self-perceived functional status 2–4 years post-stroke. We further hypothesize that when the dominant side is affected by the stroke, the correlations will be higher than when the non-dominant side is affected because of the greater effect this would have on everyday function.

## 2 Methods

### 2.1 Participants

Twenty-six (26) Participants took part in this cross-sectional study. Inclusion criteria were: (a) first unilateral stroke (ischemic or hemorrhagic) (Murphy et al., 2011), (b) age 18–85 years (Kamper et al., 2002), (c) Mini-Mental State Examination (MMSE) score ≥ 24/30 (Anderson et al., 2018) for participants >65, or MOCA score > 23 for participants <65, (d) Fugl-Meyer Upper Extremity assessment (FMA) score 16–60: a validated four-level classification of the FMA was used in order to include only patients who have at least some movement ability in the affected arm (Woytowicz et al., 2016). Please see Table 1 for further description of the participants.

**Table 1:** Demographic information describing the participants.

**Baseline information**	**Mean ± SD or range**
Age	56.8 ± 12.6
FMA score	39 (17–58)
ARAT score	36.5 (6–56)
MMSE score	24 (24–28)
MOCA score	25 (23–29)
	**Number of participants**
Gender (M/W)	13/13
Lesion type (I/H)	21/5
Affected brain side (D/ND)	15/11

M/W, men/women; I/H, ischemic/hemorrhagic; D/ND, dominant/non-dominant.

Stroke patients with additional neurological or musculoskeletal problems (such as Parkinson’s disease, unilateral neglect, Pusher syndrome and apraxia), with severe vision or sensory deficits affecting upper limb movements, or with aphasia affecting understanding of simple instructions, were excluded (Levin et al., 2016).

Due to technical difficulties (software malfunction) which led to loss of data, the correlations including kinetic measurements were conducted on a sample size of 17 participants, with median FMA scores of 33 (18–54) and median ARAT scores of 28 (13–44). In addition, the sample size for the logistic regression was 19 participants since not all participants answered the telephone interview.

### 2.2 Experimental procedure

This study is a part of a larger project, described elsewhere (Feingold-Polak et al., 2021a). It was conducted in the ambulatory unit of the Rehabilitation Center “Adi Negev” located in Israel. The Helsinki ethical committee for clinical trials approved the research (SMC-5273-2018). The kinematic and kinetic assessment, clinical tests and self-evaluation questionnaires that were conducted when participants entered the study were used in the current study. In addition, 2 years after the end of the clinical trial, a telephone interview was conducted with participants regarding the rehabilitation of their affected hand, the extent to which their affected hand was used, as well as their overall rehabilitation.

### 2.3 Assessment measures

#### 2.3.1 Self-evaluation questionnaires

*Stroke impact scale (SIS):* The SIS is a quality-of-life questionnaire that assesses changes in impairment, activities, and participation post-stroke. It was designed to track change over time and to be administered in both clinical and research fields. The SIS contains 59 questions divided into 8 domains. The last question of the questionnaire aims to assess the individual’s perceived overall recovery. Responders are asked to rate their recovery on a scale between 0 and 100, with ‘0’ indicating no recovery, and ‘100’ indicating full recovery. The SIS is highly reliable (ICC = 0.7–0.92 in all domains except for the emotional domain), valid (>0.8 in the hand function, mobility and ADL\IADL domains), and sensitive to change (Duncan et al., 1999, 2003).

*Upper Extremity Motor Activity Log (UE MAL)*: The UE MAL is a questionnaire designed to estimate how much and how well post-stroke patients use their affected hand. It includes 30 questions regarding daily activities performed using the upper extremity. Each question is rated on two scales: amount-of-use scale (AS) and how-well scale (HWS). The UE MAL HWS is reliable (0.91) and valid (0.7) (Uswatte et al., 2005).

#### 2.3.2 Clinical tests

*Fugl-Meyer Assessment Upper Extremity (FMA UE)*: The FMA UA is a 33-items clinical test which examines upper extremity motor ability, including reflex, grasp and coordination. Each item of the test is ranked on a scale of 0 to 2 (0, cannot perform; 1, performs partially; 2, performs fully). The FMA UE is highly reliable (ICC > 0.95) and valid (*r* = 0.94–0.95) (Platz et al., 2005; Lundquist and Maribo, 2017).

*Action Reach Arm Test (ARAT)*: The ARAT is designed to assess upper-extremity activity limitations. It is divided into four subscales: grasp., grip, pinch and gross movement, and includes 19 items. Each item is ranked between 0 and 3, while 0 indicates inability to perform the task and 3 indicates normal performance. The ARAT is highly reliable (ICC = 0.98–0.99) and valid (*r* = 0.91–0.94) (Lang et al., 2013).

#### 2.3.3 Kinematic and kinetic evaluation

The full details of the experimental design can be found in (Kashi et al., 2020; Feingold-Polak et al., 2021a,b). Briefly, the kinematic and kinetic measurements were conducted while participants sat on a chair with back support but without armrests, facing a 75-cm high table. The participants were instructed to reach toward a cup on the table at a self-selected speed once the “beep” sound was heard. After reaching the cup at their own pace, they grasped it, lifted it and placed it on top of a block of five centimeters high positioned on the table (Feingold-Polak et al., 2021a,b). There was a horizontal alignment between the cup and their reaching arm.

To avoid excessive trunk movement during the reach movement, the cup was placed at an arm’s distance, measured from the lateral acromion to the radial styloid process. In the reach movement, participants were instructed to avoid bending the trunk as much as possible, however the movement of the trunk was not restricted. Two different weight cups were used for the reach and grasp movements: an empty cup (273 g) and a cup filled with water (443 g). Cups were capped to prevent spills and participants were informed whether they were empty or full. For the test, a custom-built 3D-printed cup was used. There is a diameter of 6.5 cm at the base of the cup, where it is gripped by the participants; the height of the cup is 20.3 cm. An embedded 3D force sensor (Nano25-E Transducer, ATI Industrial Automation, INC) was used to measure grip forces. Force sensors sample data at a frequency of 100 Hz. Every measurement trial (each reach movement) requires calibration of the sensor force. The force sensor records the summed grip force applied to the cup. The TRIO v120 (Optitrack) motion-capture system was used to record the movement of the arm. The raw data were then used for analysis using custom-written code in MATLAB.

##### 2.3.3.1 Kinematic and kinetic variables

*Jerk:* Jerk was calculated and normalized to represent the smoothness of the hand movement, as described by Buma et al. (2016). The jerk was normalized by the movement duration, and by the distance traveled. To meet the assumptions of normality, normalized jerk (NJ) values were log-transformed (Buma et al., 2016; Feingold-Polak et al., 2021a,b).

*Mean and Maximal Velocity*: Mean and maximal velocity were determined from the tangential velocity traces of the wrist markers (Feingold-Polak et al., 2021a,b).

*Force-to-Time Ratio:* We calculated the force-to-time ratio by dividing the mean force (in Newtons) during a movement by the duration of the movement in seconds (Feingold-Polak et al., 2021a,b). By calculating how much force is applied per time unit, this measure reflects the efficiency of force regulation, which we previously reported to be impaired in individuals post stroke (Feingold-Polak et al., 2021a,b); It may reflect the individual’s ability to anticipate the amount of force they will have to apply before they lift an object (Feingold-Polak et al., 2021a,b).

*Number of Force Peaks (NFP):* The number of force peaks that occurred during the movement. NFP was calculated as follows: (i) We calculated FT, which is 5% of the maximum force exerted during the movement; (ii) We identified all local maxima (peaks) and local minima in the force trace of that movement; (iii) Force peaks were defined as peaks with a value greater than [FT + the preceding local minimum value] (Feingold-Polak et al., 2021a,b). NFP reflects the variability in force control, thus reflecting the individual’s ability to produce an efficient, consistent force regulation during a movement (Feingold-Polak et al., 2021a,b).

##### 2.3.3.2 Self-reported functional status 2–4 years after the stroke

We contacted the participants of this study by phone 2–4 years following their stroke, and asked them the last question from the SIS questionnaire (“On a scale of 0 to 100, with 100 representing full recovery and 0 representing no recovery, how much have you recovered from your stroke?”). Their responses were transformed into a binary response (0/1) using the cutoff of a score of 5 out of 10 (a score of 1–5 corresponded to “0”, and a score of 6–10 corresponded to “1”) in order to simplify the interpretation of the results.

### 2.4 Statistical analysis

The statistical data analyses were conducted using IBM Statistical Package for Social Sciences (SPSS), version 29.0 and a custom-written script in MATLAB software (Mathworks, MA, v.R2018b). Descriptive statistics have been used to summarize the demographic information as well as the scores for the FMA-UE and the ARAT. The normality of the variables was tested using Shapiro–Wilk’s test. A heatmap was constructed to assess the correlation between kinematic and kinetic variables, SIS, MAL UE, FMA and ARAT scores. Another heatmap was constructed to assess the correlations between the different measures, split into dominant and non-dominant brain side stroke. Dominant means that the dominant side of the body prior to the stroke is the side that was mostly impaired by the stroke, and non-dominant means that the non-dominant side of the body prior to the stroke is the side that was mostly impaired by the stroke. The colors of the heatmap were coded based on spearman’s correlation coefficient. Yet another heatmap was constructed to assess the correlations between the different measures, split into a group of dominant side stroke and a group of non-dominant side stroke. The strength of correlation coefficients was interpreted as 0.00–0.25 (very low), 0.26–0.49 (low), 0.50–0.69 (moderate), 0.70–0.89 (high) and 0.90–1.00 (very high) (Schober et al., 2018). The spearman’s correlations which included kinetic variables were conducted on a sample size of 17 participants.

A logistic regression was used to evaluate the ability of the different assessment measures to predict the participants’ self-reported rehabilitation 2–4 years after the stroke. We computed an AUC based on the logistic regression to compare the prognostic ability of the measures that were found to significantly predict the self-reported rehabilitation of the participants.

A sample size estimation was not performed in this study as it was part of a larger project, which was a pilot study.

## 3 Results

The outcome measures we recorded are listed in Table 2.

**Table 2:** Measures of central tendency and variability for all assessment measures.

	**Assessment measure**	**Mean or median**	**±SD or range**
**Clinical tests**	FMA	39	17–58
	ARAT	36.5	6–56
**Kinematic variables**	Jerk	4.6	**±**0.8
	Mean velocity (mm/s)	156.3	**±**56.4
	Maximal velocity (mm/s)	580.7	**±**180.4
**Kinetic variables**	Force-to-time ratio	1.2	0.3–5.2
	Number of force peaks	0.8	0–4.5
**PROMS**	MAL AS	1.8	0–4.8
	MAL HWS	1.7	0–4.4
	SIS hand force	50	25–81.3
	SIS ADL	55	11–86
	SIS hand function	34.4	0–68.8

### 3.1 Correlations between kinematic and kinetic metrics, clinical tests, and self-evaluation questionnaires

#### 3.1.1 Hand kinematics

We found the mean and the maximal hand velocity to be correlated with the self-evaluation questionnaires and with the clinical tests, more than other kinematic variables (see Table 3 and Please see Supplementary Table S1 for a correlation matrix with the *p*-values and confidence intervals).

**Table 3:** Correlation heatmap of kinematic and kinetic variables, clinical tests and self-evaluation questionnaires.

	FMA	ARAT	MAL AS	MAL HWS	SIS hand force	SIS ADL	SIS hand function
FMA	1	**0.68**	**0.65**	**0.69**	0.14	0.24	**0.43**
ARAT	**0.68**	1	**0.78**	**0.79**	0.16	0.26	**0.5**
Jerk	**0.49**	**0.64**	**0.49**	**0.53**	0.25	**0.45**	**0.58**
Mean Velocity	**0.48**	**0.59**	**0.77**	**0.79**	**0.41**	**0.48**	**0.53**
Maximal Velocity	0.35	0.19	**0.52**	**0.53**	**0.47**	**0.4**	0.3
Force-to-Time Ratio	0.43	0.4	0.48	0.46	0.35	0.44	**0.52**
Number of Force Peaks	**0.72**	**0.51**	**0.54**	0.46	0.34	0.26	0.38

The heatmap is based on spearman’s correlation analysis between kinematic and kinetic variables, clinical tests (FMA, ARAT) and self-evaluation questionnaires (MAL AS, MAL HWS, SIS hand force, SIS ADL and SIS hand function). Significant correlations (*p* < 0.05) are marked in bold. Darker colors mean stronger correlations.

##### 3.1.1.1 Mean velocity

Mean velocity showed low [*r*(24) = 0.486, *p* = 0.012] correlation with FMA, moderate [*r*(24) = 0.592, *p* = 0.001] correlation with ARAT, high [*r*(23) = 0.776–0.79, *p* < 0.001] correlation with both MAL scales, moderate [*r*(24) = 0.531, *p* = 0.005] correlation with SIS hand function scale, and low [*r*(24) = 0.412–0.481, *p* = 0.036–0.013] correlation with SIS hand force scale and SIS ADL scale.

##### 3.1.1.2 Max velocity

Maximal velocity showed moderate [*r*(23) = 0.521–0.531, *p* = 0.006–0.008] correlation with both MAL scales, and low [*r*(24) = 0.403–0.475, *p* = 0.014–0.041] correlation with SIS hand force scale and SIS ADL scale.

#### 3.1.2 Correlations between self-evaluation questionnaires, kinematics and kinetics, and clinical tests

We found that sections regarding hand function, hand force and general ADL of the self-evaluation questionnaires correlated with kinematic variables. However, only questionnaires that focus on hand function correlated with clinical tests [see Table 3 for correlation heatmap of kinematic and kinetic variables, clinical tests and self-evaluation questionnaires].

##### 3.1.2.1 Self-evaluation questionnaires and kinematics and kinetics

Both MAL scales showed moderate [*r*(23) = −0.499–0.535, *p* = 0.006–0.011] inverse correlation with jerk and maximal velocity, and high [*r*(23) = 0.776–0.79, *p* < 0.001] correlation with mean velocity. Both SIS hand force scale and SIS ADL scale showed low [*r*(24) = 0.403–0.481, *p* = 0.013–0.041] correlation with mean and maximal velocity. SIS ADL scale also showed low [*r*(24) = −0.453, *p* = 0.02] inverse correlation with jerk.

SIS hand function scale showed moderate [*r*(24) = 0.531–(−0.587), *p* = 0.002–0.005] correlation with jerk and mean velocity. It also showed moderate [*r*(15) = 0.52 *p* = 0.032] correlation with force-to-time ratio.

##### 3.1.2.2 Self-evaluation questionnaires and clinical tests

SIS hand function scale showed low [*r*(24) = 0.431, *p* = 0.028] correlation with FMA and moderate [*r*(24) = 0.504, *p* = 0.009] correlation with ARAT.

Both MAL scales showed moderate [*r*(23) = 0.657–0.698, *p* < 0.001] correlation with FMA and high [*r*(23) = 0.786–0.797, *p* < 0.001] correlation with ARAT.

#### 3.1.3 Hand kinetics

We found the self-evaluation questionnaires and the clinical tests to be correlated with hand kinetic metrics force-to-time ratio and number of force peaks. Number of force peaks showed high [*r*(15) = −0.726, *p* < 0.001] inverse correlation with FMA, moderate [*p*(15) = −0.512, *p* = 0.036] inverse correlation with ARAT and moderate [*r*(14) = −0.542, *p* = 0.030] inverse correlation with MAL AS. Force-to-time ratio showed moderate (0.520) correlation with SIS hand function scale (see Table 3).

#### 3.1.4 Correlations between self-evaluation questionnaires, kinematics and kinetics, and clinical tests, split into dominant and non-dominant brain side stroke

When split into dominant and non-dominant brain side stroke, we found stronger correlations between the different measures in the dominant affected brain side group. In addition, this group had a higher number of significant correlations between the different measures. For further information please see Table 4.

**Table 4:** Correlation heatmap of kinematic and kinetic variables, clinical tests and self-evaluation questionnaires split into dominant vs. non-dominant brain side stroke.

Non-dominant brain side									Dominant brain side								
	FMA	ARAT	MAL AS	MAL HWS	SIS hand force	SIS ADL	SIS hand func.	General func.		FMA	ARAT	MAL AS	MAL HWS	SIS hand force	SIS ADL	SIS hand func.	General func.
FMA	1	0.51	**0.73**	**0.81**	0.12	0.21	0.37	0.09	FMA	1	**0.7**	**0.68**	**0.67**	0.1	0.3	0.4	**0.55**
ARAT	0.51	1	**0.63**	0.59	0.03	0.31	0.59	0.19	ARAT	**0.7**	1	**0.81**	**0.86**	0.24	0.13	0.4	0.28
Jerk	**0.63**	**0.71**	0.33	0.38	0.21	0.08	0.16	0.29	Jerk	0.45	**0.53**	**0.58**	**0.61**	**0.52**	**0.54**	**0.6**	**0.66**
MeanV	0.58	0.29	**0.67**	**0.73**	0.25	0.26	0.11	0.29	MeanV	0.31	0.57	**0.71**	**0.73**	0.47	0.44	**0.58**	**0.66**
MaxV	0.11	0.21	0.3	0.33	0.31	0.2	0.08	0.48	MaxV	0.34	0.33	**0.54**	**0.54**	**0.53**	0.29	0.4	**0.57**
FtTR	0.56	0.3	0.2	0.2	0.3	0.56	0.15	–	FtTR	0.56	0.37	**0.62**	**0.58**	**0.7**	**0.59**	**0.58**	0.41
NoFP	0.8	0.35	0	0	0.05	0.15	0.35	–	NoFP	**0.67**	0.51	**0.61**	0.54	0.44	0.3	0.34	0.43
General func.	0.09	0.19	0.48	0.29	**0.89**	**0.89**	0.5	1	General func.	**0.55**	0.28	0.53	0.45	0.37	0.33	0.39	1

The heatmap is based on spearman’s correlation analysis between kinematic and kinetic variables, clinical tests (FMA, ARAT) and self-evaluation questionnaires (MAL AS, MAL HWS, SIS hand force, SIS ADL and SIS hand function). The general function column relates to the general function question asked in the telephone interview. Significant correlations (*p* < 0.05) are marked in bold. Darker colors mean stronger correlations.

### 3.2 Prediction of self-reported general function 2–4 years post-stroke

The question regarding general function, asked in the telephone interview, was transformed into a binary response. We used logistic regressions to estimate the probability of a positive (‘1’) or negative (‘0’) general function outcome based on the SIS hand force scale, mean velocity, and maximal velocity, since those were the only variables which significantly correlated with the general function question. Due to the small number of participants, the lateralization of the stroke was not related to in these analyses.

**Figure 1:**
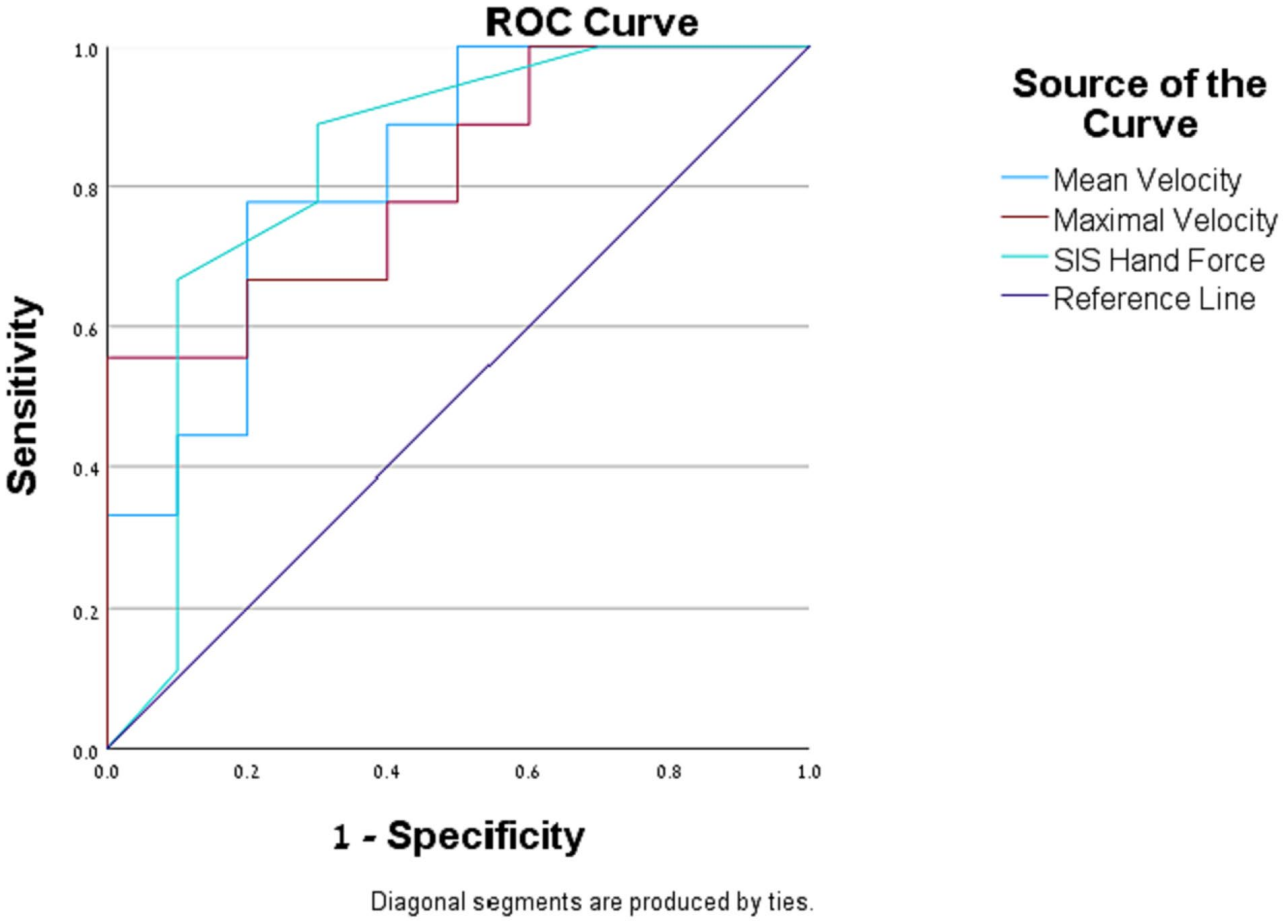
Logistic regression. Receiver operating characteristic (ROC) curves of the assessment measures that were found to be significantly correlated with the self-reported general function question.

We found that the odds that an individual will assess their general function as being above 50% of their function before the stroke: (1) increased by 8.1% (95% CI [0.001, 0.16]) for a one-unit increase in SIS hand-force scale; (2) increased by 2.6% (95% CI [0.002, 0.05]) for a one-unit increase in the hand’s mean velocity (mm/s); (3) increased by 1% (95% CI [0.00, 0.02]) for a one-unit increase in the hand’s maximal velocity mm/s. The AUCs of SIS hand-force scale, mean velocity and maximal velocity were 0.828, 0.822, and 0.811, respectively (Figure 1).

All other assessment measures did not significantly predict the participant’s self-reported general function.

## 4 Discussion

Our goal in this study was to explore the importance of self-evaluation questionnaires as part of clinical patient evaluation, and to determine which functional kinematic and kinetic variables correlate with patients’ clinical assessment and self-perceived recovery. The importance of implementing kinematic and kinetic evaluations in stroke research was emphasized by the Stroke Recovery and Rehabilitation Roundtable (SRRR), since those are the only assessment measures which can evaluate behavioral restitution (Bernhardt et al., 2017). Therefore, kinematic and kinetic measures were strongly advised to be considered as outcome measures in future trials (Kwakkel et al., 2017). We had four main findings in this study: First, the self-evaluation questionnaires sections regarding hand function, hand force and general ADL were correlated with kinematic variables, while only questionnaires sections focusing on hand function correlated with clinical tests. Second, self-evaluation questionnaires and clinical tests showed the strongest correlations with mean and maximal hand velocity, more so than other kinematic variables. Our third main finding was that hand kinetic metrics force-to-time ratio and number of force peaks were correlated with self-evaluation questionnaires and clinical tests. Our final key finding was that self-reported general function 2–4 years after a stroke was predicted by the SIS hand force domain, mean velocity, and maximal velocity.

### 4.1 Correlation between clinical tests and kinematics

We found the ARAT and the FMA clinical tests to correlate with jerk and mean velocity. This is in line with previous literature, where the ARAT and the FMA were found to correlate with kinematics; Specifically with mean velocity, maximal velocity, and jerk (Lang et al., 2006; Li et al., 2015; Nijenhuis et al., 2018).

### 4.2 Correlation between self-evaluation questionnaires and kinematics

#### 4.2.1. UE-MAL and kinematics

The UE-MAL has been correlated in the past with kinematics, such as trunk displacement, number of velocity peaks and number of movement units (Nijenhuis et al., 2018; Adans-Dester et al., 2020). Our findings, that UE-MAL correlates with mean velocity, maximal velocity and jerk have not been previously reported (Nijenhuis et al., 2018; Adans-Dester et al., 2020). The correlations between UE-MAL and the various kinematic variables found in our study emphasize the strength of this evaluation tool, and the importance of using it both in clinical practice and in future research. The strong correlations found between UE-MAL and kinematics may be because some of the questions ask participants to rate their hand’s motor ability; For example, according to the UE-MAL manual, a score of 2 on the HWS means that “The weaker arm was of some use during that activity but needed some help from the stronger arm or moved very slowly or with difficulty.”

#### 4.2.2. SIS and kinematics

The SIS *hand-function* domain has previously been found to be correlated with movement time and amount of movement units (Thrane et al., 2018). This finding is in line with our finding, according to the SIS *hand-function* correlates with the kinematic variables *mean velocity* and *jerk*. In addition, we found correlations between kinematic variables and the SIS domains of *hand-force* and *ADL,* which have not been previously reported. The correlation between the kinematics and the three SIS domains demonstrates that the individual’s impairment – as reflected by the kinematic analysis of their hand – has a major influence on how they perceive their impairment, function and participation.

We found that all self-evaluation questionnaires correlate with kinematic variables, which are sensitive and accurate assessment measures. However, only questionnaires that focus on *hand function* correlate with clinical tests. These findings demonstrate the limitation of solely evaluating post-stroke patients with clinical tests, which leaves out important aspects of the patients’ rehabilitation.

### 4.3 Hand velocity

We found the *mean* and the *maximal hand velocity* to be more highly correlated with the *self-evaluation questionnaires* and with the *clinical tests* than other kinematic variables. This finding adds to existing literature on the centrality of hand velocity as an outcome measure in stroke research. For example, hand velocity has been previously reported to be highly sensitive to learning effect; parameters that demonstrate a persistent learning effect over sessions can be used to assess the effectiveness of different therapeutic interventions on learning processes post-stroke (Brihmat et al., 2020). In a systematic review which investigated clinimetrics of commonly-used kinematic metrics, movement time was reported as a valid measurement, supported by high-quality evidence (Schwarz et al., 2019). Hand velocity is indeed a key component of most functions of the hand, both unilateral functions (such as reach-to-grasp tasks or writing) and bilateral functions (such as dressing or shoe tying), and related to response time (Banina et al., 2017). When hand movement is perceived as too slow or when the involved hand is slower than the unaffected hand in bilateral functions, it can negatively affect the function and the use of the hand in everyday life, eventually affecting participation. The findings we report on hand velocity’s correlations with the self-evaluation questionnaires, and with the clinical tests, can therefore also help direct clinicians when working with patients: focusing on hand velocity as part of the rehabilitation plan might influence the patients’ hand function and self-perceived hand function, hand force and general ADL.

### 4.4 Functional kinetics

We found that number of force peaks correlated with FMA, ARAT and MAL AS and that force-to-time ratio correlated with the SIS hand function scale. Examination of correlations between functional kinetics and clinical tests and self-evaluation questionnaires has not been previously reported in the literature. Previous studies assessed different parameters of hand force and their correlations with clinical tests and self-evaluation questionnaires (Krebs et al., 2014; Plantin et al., 2022), but this is the first time, to the best of our knowledge, that kinetics are evaluated during a *functional* task. The functionality of a kinetic measurement task is highly important since only functional, everyday tasks can represent the real-life activity of an individual (Bayona et al., 2005; Hubbard et al., 2009). Therefore, it is impossible to deduce from general hand-strength tests on the patients’ actual functional abilities (Winstein et al., 2016). Force-to-time ratio and number of force peaks measure the force regulation ability of the individual, which is related to their functional ability. The number of force peaks reflects the variability of force an individual applies on a grasped object during a reach-to-grasp task, and it was first described by Feingold-Polak et al. (2021a,b).

In addition to the advantages associated with using functional kinetics as outcome measures, it is important to be mindful of the possibility of data loss due to technical difficulties.

### 4.5 The importance of self-evaluation questionnaires in the clinical field

We found that all self-evaluation questionnaires correlate with kinematic variables, which are the most sensitive and accurate assessment measures. However, only questionnaires that focus on *hand function* correlate with clinical tests. We suggest that this finding can explain the blind spot presented in previous studies – it indicates that clinical tests cannot tell the full story of the patient’s rehabilitation progression, and highlights the importance of using self-evaluation questionnaires in addition to clinical tests. Our findings strengthen recent evidence which suggests that patient perspective should be an inseparable part of stroke rehabilitation assessment (Luker et al., 2015). Self-evaluation questionnaires should be integrated into post-stroke evaluation, both in the clinical field and in research. They can be used to measure the improvement of a specific individual and can help formulate directions for treatment of an entire diagnostic group (Winstein and Varghese, 2018). In addition, our findings show the strong relationship between the patient’s impairment and participation.

While self-evaluation questionnaires are valuable assessment measures, one of their major drawbacks is their participation rate: Glimmerveen et al. (2023) found than only 45% of stroke patients who were discharged from hospital complied with the hospital’s request to fill out self-evaluation questionnaires. In addition, the patients who filled out the questionnaires were less impaired than those who did not fill them out. It is thus important to think of creative ways to provide accessibility and engage patients and caregivers in filling out self-evaluation questionnaires.

Self-evaluation questionnaires relate mostly to functions the patient preforms at home. In a hospitalized rehabilitation setting, the patient has less opportunities to experience a variety of daily functions; therefore, the existing self-evaluation questionnaires might not be suitable for every stage of the rehabilitation process. Self-evaluation questionnaires that can adapt to the different rehabilitation stages could help achieve a more accurate picture of the patient’s functional status.

### 4.6 Prediction of self-reported general function several years post-stroke

Of all outcome measures used in this study, only three were able to significantly predict the participants’ self-reported general function 2–4 years after the stroke: *SIS hand force scale*, *hand mean velocity* and *hand maximal velocity*. This finding emphasizes the limitation of clinical tests in long-term prediction of function. It supports the conclusion of a previous study, which found that the clinical evaluation 1 year post-stroke does not reflect the amount of daily use of the affected hand (Rand and Eng, 2015). Our conclusion also underlines the potential of the SIS hand force domain and hand velocity kinematic measurements as important outcome measures in post-stroke rehabilitation assessment, and shows the strong link between the impairment, represented by the kinematics, and the participation, represented by the self-evaluation questionnaires.

### 4.7 Brain side dominance

When we split the participants into dominant and non-dominant affected brain side groups, we found stronger and a higher number of statistically significant correlations between the different assessment measures in the dominant affected brain side group. The main difference we found between the two groups was in the correlations between the kinematic and kinetic measures, which represent the impairment of the hand, and the self-evaluation questionnaires, which represent the activity and participation of the individual. An explanation to this mismatch could be that when the affected hand is not the dominant hand, the effect of the hand’s impairment on the overall function of the individual is less significant. The existing literature also supports this explanation – the dominance of the affected brain side after stroke has not been found to have a significant effect on function and quality of life (Nam et al., 2014; Lee et al., 2020). However, the dominance of the affected hand does have an impact on the impairment level; individuals with a dominant brain side stroke show less impairment compared to those with a non-dominant brain side stroke (Harris and Eng, 2006). In addition, Feingold-Polak et al. (2021a,b) found that when the non-dominant hand was used, too much force was recruited for completing the task, while when the dominant hand was used, too little force was recruited for task completion, independent of stroke severity.

### 4.8 The main contributions of this paper, and the importance of our findings

The three main contributions of our study are: first, our findings emphasize the importance of using self-evaluation questionnaires as an evaluation measure in post-stroke patients, in addition to clinical tests. Second, we found that hand velocity is an accurate, robust kinematic measurement and should be considered as an outcome measure in future studies and in clinical practice. Third, the correlation between functional kinetics and common evaluation tools has not yet been reported. Our results suggest that *Number of Force Peaks* and *Force-to-Time Ratio* are two force-regulation variables that should be considered as outcome measures in future studies.

## 5 Conclusion

Self-evaluation questionnaires are the only evaluation tools that consider the patient’s self-perspective; The completion of a questionnaire can assist the patient in reflecting on their rehabilitation process and in strengthening their sense of achievement. We found that the questionnaires MAL and SIS correlate with kinematics and kinetics of the affected hand. We also found that the self-evaluation questionnaires we examined also partially correlate with the ARAT and the FMA. We therefore argue that self-evaluation questionnaires should be used in the clinical assessment process. On the one hand, they may indicate the individuals’ functional status, and on the other, a mismatch between them and the clinical scores or the kinematic and kinetic measures, could inform an individual’s rehabilitation plan: The mismatch between the evaluations may have an emotional, cognitive, or motivational basis; this should be identified and treated accordingly.

## 6 Study limitations

The limitation of our study concerns the relatively small sample-size (*N* = 26). Moreover, the kinetic measurements for nine of the 26 participants are missing due to technical difficulties; therefore, the analyses which involve kinetic measurements were conducted on an even smaller number of participants (*N* = 17). In addition, after splitting the participants into dominant and non-dominant brain side stroke groups, there were fewer participants in each correlation analysis (*N* = 15 and *N* = 11, respectively). The logistic regressions were also conducted on a smaller number of participants due to missing data (*N* = 19).

## Data availability statement

The raw data supporting the conclusions of this article will be made available by the authors, without undue reservation.

## Ethics statement

The studies involving humans were approved by The Sheba Medical Center Helsinki committee. The studies were conducted in accordance with the local legislation and institutional requirements. Written informed consent for participation in this study was provided by the participants’ legal guardians/next of kin.

## Author contributions

RB: Conceptualization, Data curation, Investigation, Writing – original draft. RF-P: Writing – review & editing, Conceptualization, Methodology. DO: Formal analysis, Writing – review & editing. IK: Writing – review & editing. SL-T: Methodology, Supervision, Writing – review & editing.

## Supplementary material

SUPPLEMENTARY TBALE S1 Correlation heatmap of kinematic and kinetic variables, clinical tests and self-evaluation questionnaires. The heatmap is based on spearman’s correlation analysis between kinematic and kinetic variables, clinical tests (FMA, ARAT) and self-evaluation questionnaires (MAL AS, MAL HWS, SIS hand force, SIS ADL and SIS hand function). Significant correlations (*p* < 0.05) are marked in bold.
