# Corrigendum: Correlation between kinetic and kinematic measures, clinical tests and subjective self-evaluation questionnaires of the affected upper limb in people after stroke

**DOI:** 10.3389/fnins.2023.1352007

**Published:** 2023-12-18

**Authors:** Ronnie Baer, Ronit Feingold-Polak, Daniel Ostrovsky, Ilan Kurz, Shelly Levy-Tzedek

**Affiliations:** ^1^Recanati School for Community Health Professions, Department of Physical Therapy, Faculty of Health Sciences, Ben-Gurion University of the Negev, Be'er Sheva, Israel; ^2^Herzog Medical Center, Jerusalem, Israel; ^3^Clinical Research Center, Soroka University Medical Center, Ben-Gurion University of the Negev, Be'er Sheva, Israel; ^4^Zelman Center for Neuroscience, Ben-Gurion University of the Negev, Beer-Sheva, Israel; ^5^Freiburg Institute for Advanced Studies (FRIAS), University of Freiburg, Freiburg, Germany

**Keywords:** stroke, kinematics, kinetics, clinical test, self-evaluation questionnaires

In the published article, there was an error in the Funding statement. The statement mistakenly indicted that no financial support was received for the research, authorship, and/or publication of the article. The correct Funding statement appears below.

