# Supplementary material for: Correlation between kinetic and kinematic measures, clinical tests and subjective self-evaluation questionnaires of the affected upper limb in people after stroke
Source: Front Neurosci. 2023 Dec 4;17:1264513. doi: 10.3389/fnins.2023.1264513 (PMC10765579; doi:10.3389/fnins.2023.1264513)
Supplement: SUPPLEMENTARY TBALE S1 — Correlation heatmap of kinematic and kinetic variables, clinical tests and self-evaluation questionnaires. The heatmap is based on spearman’s correlation analysis between kinematic and kinetic variables, clinical tests (FMA, ARAT) and self-evaluation questionnaires (MAL AS, MAL HWS, SIS hand force, SIS ADL and SIS hand function). Significant correlations (p < 0.05) are marked in bold. [file Table_1.docx]

|  | FMA | ARAT | MAL AS | MAL HWS | SIS Hand Force | SIS ADL | SIS Hand Function |
| --- | --- | --- | --- | --- | --- | --- | --- |
| FMA | **1** | **0.68** p<0.001 CI [0.38, 0.84] | **0.65** p<0.001 | **0.69** p<0.001 | 0.14 p=0.48 CI [-0.27, 0.51] | 0.24 p=0.23 CI [-0.17, 0.58] | **0.43 p=0.02 CI [0.04, 0.7]** |
| ARAT | **0.68** p<0.001 CI [0.38, 0.84] | **1** | **0.78** p<0.001 | **0.79** p<0.001 | 0.16 p=0.41 CI [-0.24, 0.53] | 0.26 p=0.2 CI [-0.15, 0.59] | **0.5** **p=0.009 CI [0.13, 0.75]** |
| Jerk | **0.49 p=0.01 CI [-0.74, -0.12]** | **0.64 p<0.001 CI [-0.83, -0.33]** | **0.49 p=0.01 CI [-0.75, -0.11]** | **0.53 p=0.006 CI [-0.77, -0.16]** | 0.25 p=0.2 CI [-0.59, 0.15] | **0.45 p=0.02 CI [-0.72, -0.06]** | **0.58** **p=0.002 CI [-0.79, -0.24]** |
| Mean Velocity | **0.48** **p=0.012 CI [0.11, 0.74]** | **0.59 p=0.001 CI [0.25, 0.8]** | **0.77 p<0.001 CI [0.54, 0.89]** | **0.79 p<0.001** | **0.41 p=0.036 CI [0.17, 0.69]** | **0.48 p=0.13 CI [0.1, 0.73]** | **0.53 p=0.005 CI [0.16, 0.76]** |
| Maximal Velocity | 0.35 p=0.07 CI [-0.05, 0.65] | 0.19 p=0.34 CI [-0.22, 0.54] | **0.52 p=0.008 CI [0.14, 0.76]** | **0.53 p=0.006 CI [0.16, 0.77]** | **0.47 p=0.014 CI [0.09, 0.73]** | **0.4 p=0.041 CI [0.007, 0.69]** | 0.3 p=0.12 CI [-0.1, 0.62] |
| Force-to-Time Ratio | 0.43 p=0.07 CI [-0.06, 0.76] | 0.4 p=0.1 CI [-0.11, 0.74] | 0.48 p=0.055 CI [-0.02, 0.79] | 0.46 p=0.068 CI [-0.05, 0.78] | 0.35 p=0.15 CI [-0.16, 0.72] | 0.44 p=0.07 CI [-0.06, 0.76] | **0.52 p=0.03** **CI [0.03, 0.8]** |
| Number of Force Peaks | **0.72 p<0.001 CI [-0.89, -0.36]** | **0.51 p=0.03 CI [-0.8, -0.02]** | **0.54 p=0.03 CI [-0.82, -0.04]** | 0.46 p=0.06 CI [-0.78, 0.05] | 0.34 p=0.17 CI [-0.71, 0.18] | 0.26 p=0.3 CI [-0.66, 0.26] | 0.38 p=0.13 CI [-0.73, 0.13] |
